# Using Patient Pathway Analysis to Design Patient-centered Referral Networks for Diagnosis and Treatment of Tuberculosis: The Case of the Philippines

**DOI:** 10.1093/infdis/jix391

**Published:** 2017-11-06

**Authors:** Celine Garfin, Mariquita Mantala, Rajendra Yadav, Christy L Hanson, Mike Osberg, Aaron Hymoff, Julia Makayova

**Affiliations:** 1 Department of Health of the Philippines, National Tuberculosis Program; 2 Technical Assistance to Country project; 3 World Health Organization, Office of the Philippines, Manila; 4 Macalester College, St. Paul, Minnesota; 5 Bill and Melinda Gates Foundation,; 6 Linksbridge, Seattle, Washington.

**Keywords:** tuberculosis, patient pathway analysis, MDR-TB, care seeking, diagnosis

## Abstract

**Background:**

Tuberculosis (TB) is the 8th leading cause of death in the Philippines. A recent prevalence survey found that there were nearly 70% more cases of tuberculosis than previously estimated. Given these new data, the National TB Program (NTP), operating through a decentralized health system, identified about 58% of the estimated new drug-sensitive (DS) TB patients in 2016. However, the NTP only identified and commenced treatment for around 17% of estimated new drug-resistant patients. In order to reach the remaining 42% of drug-sensitive patients and 83% of drug-resistant patients, it is necessary to develop a better understanding of where patients seek care.

**Methods:**

National and regional patient pathway analyses (PPAs) were undertaken using existing national survey and NTP data. The PPA assessed the alignment between patient care seeking and the availability of TB diagnostic and treatment services.

**Results:**

Systemic referral networks from the community-level Barangay Health Stations (BHSs) to diagnostic facilities have enabled more efficient detection of drug-sensitive tuberculosis in the public sector. Approximately 36% of patients initiated care in the private sector, where there is limited coverage of appropriate diagnostic technologies. Important differences in the alignment between care seeking patterns and diagnostic and treatment availability were found between regions.

**Conclusions:**

The PPA identified opportunities for strengthening access to care for all forms of tuberculosis and for accelerating the time to diagnosis by aligning services to where patients initiate care. Geographic variations in care seeking may guide prioritization of some regions for intensified engagement with the private sector.

The Sustainable Development Goals (SDGs)—adopted in 2015—reflect a global commitment to end poverty and improve health outcomes for all [1]. With a population of 101 million, the 13th largest in the world, the Philippines plays an important role in the global progress of the SDGs [2, 3]. The Philippines has embraced universal health coverage (UHC) as its national health strategy and has one of the longest-running national health insurance schemes—PhilHealth—in the region [4].

The Philippines has made strides in its aim to reduce poverty, successfully elevating more than 10% of the population out of poverty since 1991 [4–7]. Still, about 23 million Filipinos live in poverty and approximately 38% of the population remains vulnerable to poverty, with incomes only marginally above the national poverty line [8, 9]. Out-of-pocket health spending accounted for more than 50% of total health spending in 2015 [10].

Tuberculosis (TB), a disease inextricably linked with poverty, continues to threaten both individual and national economic development in the Philippines [11]. Despite halving TB mortality and prevalence between 1990 and 2012, tuberculosis remains the 8th leading cause of death in the country [4, 8]. In 2016, the Philippines had an estimated TB prevalence of 554 per 100,000 population, including around 30,000 patients with rifampicin resistance or multi-drug resistant tuberculosis (MDR-TB) [12]. In 2016, the National Tuberculosis Program (NTP) notified 330,000 patients, or 58% of the estimated incidence [12–14]. However, the NTP estimates that real number of notified cases may be only about 250,000 given an over-reliance on x-ray for diagnosis and the likely high reporting of false positive cases. The detection and management of MDR-TB patients continue to be a challenge, with only 17% of all estimated MDR-TB cases treated in 2015 [12].

To reach all patients and reduce delays in diagnosis, treatment, and cure, TB services must reach patients where they seek care. The Philippines features a decentralized model of government, with 17 functional regions and 81 provinces spread across an archipelago of more than 7,100 islands [4, 5]. Healthcare is similarly decentralized, with administrative and financial responsibilities residing at the regional and local government unit (LGU) levels [4, 5]. TB care is provided at the primary health care level through the most decentralized public care centers, namely, Barangay Health Stations (BHSs) [5]. A referral system connects the BHSs with higher-level facilities for diagnostic screening, testing, and treatment initiation [14].

Given the new knowledge that prevalence is not declining as previously believed, the NTP seeks to better understand where it is missing patients and how it can accelerate the identification and successful treatment of all patients. This study provides new evidence regarding the potential service delivery gaps that may be contributing to the persistent prevalence.

## METHODS

The patient pathway analysis (PPA) methodology described in Hanson et al. [15] was used to assess the alignment between patient care seeking and the availability of TB diagnostic and treatment services. Two sources of national-level care seeking data were compared, namely, from the 2013 Demographic and Health Survey (DHS) and the 2017 National TB Prevalence Survey (NTPS) [6, 16]. Additionally, PPAs were completed subnationally for the 17 administrative regions, using the DHS data. The primary data points considered by the PPA were care initiation patterns for general illness, TB diagnostic availability, and TB treatment availability at the site of care initiation. The data sources for each are shown in Table 1. Further background on each data source is provided in the supplementary appendix to this article.

**Table 1.** Primary Data Sources

PPA component	Data source
Care seeking	2013 Demographic and Health Survey [6]
	2017 National TB Prevalence Survey [16]
TB service availability	Philippines ITIS Database (accessed 04/01/17) [17]
Health facility availability	National Health Facility Registry (accessed 04/01/17) [18]

Abbreviations: ITIS, integrated TB information system; PPA, patient pathway analysis; TB, tuberculosis.

Since each of the data sources used a different naming convention for health facilities, common categories to designate facility level were created to allow for comparison across data sources. Each individual facility was designated as public, private (formal), or private (informal).

### Health Facilities Were Defined by the Following Categories

Level 0 (L0) refers to the most basic and usually community-based care level. Level 0 services include basic triage, health information, and essential prevention and care. Services are commonly provided as an extension of facility-based care and are provided by midwives who are supervised by nurses and supported by Barangay health workers. No laboratory testing is available but L0 staff may serve as treatment supporters for TB patients and may include sputum collection or smearing stations. Examples: Barangay Health Station, outreach clinic (public); and alternative medical facility (private).

Level 1 (L1) refers to a facility that provides primary health care. Nurses, midwives, and doctors commonly provide L1 services, generally on an outpatient basis. Some basic diagnostic services and essential medicines may be available. Examples: DOTS center, Rural Health Unit (public); NGO and private clinics (private).

Level 2 (L2) refers to facilities that provide primary health care as well as specialized care. L2 facilities commonly have more extensive diagnostic and treatment options and can provide both outpatient and in-patient care. Examples: any public hospital (public), and private hospital (private).

Several data sources did not distinguish between level 2 and level 3 hospitals in the public sector. In order to calculate care seeking, coverage and access to care at the site of initial care, all hospitals were categorized as level 2. Table 2 shows a detailed mapping of health facilities from each data source.

**Table 2.** Health Facility Categorization

Data source	Health facility type (from survey)	Mapped to --->	Health facility sector	Health facility level
DHS—General illness	Provincial hospital		Public	Level 2
	Regional hospital/medical center		Public	Level 2
	District hospital		Public	Level 2
	Municipal hospital		Public	Level 2
	Rural Health Unit (RHU)/Urban Health Center		Public	Level 1
	Barangay health station (BHS)		Public	Level 0
	Mobile clinic/other public		Public	Level 0
	Private hospital/clinic		Private	Level 2
	Other private		Private	Level 1
	Private clinic		Private	Level 1
	Alternative medical		Informal Private	Level 0
	Nonmedical		Informal Private	Level 0
	Other/missing		Other	Other
National TB prevalence survey 2016	Provincial hospital/public medical center		Public	Level 2
	RHU/Urban health center/DOTS TB clinic		Public	Level 1
	Other public		Public	Level 1
	Private hospital		Private	Level 2
	Private clinic		Private	Level 1
	PPM DOTS		Private	Level 1
	Private pharmacy		Private	Level 0
	NGO clinic		Private	Level 1
	Other private		Private	Level 1
	Given by relatives/friends		Informal Private	Level 0
	Others			
ITIS - Dx service	Provincial health office		Public	Level 2
	Regional office		Public	Level 2
	City health office		Public	Level 1
	Barangay health station (BHS)		Public	Level 0
	Jail		Public	Other
	Prison		Public	Other
	Hospital		Private	Level 2
	Hospital-based		Both	Level 2
	Clinic		Both	Level 1
	Rural health unit (RHU)/Health center		Both	Level 1
ITIS - Tx service	Hospital		Both	Level 2
	Clinic		Both	Level 1
	Rural ealth unit (RHU)/Health center		Both	Level 1
	Jail		Both	Other
National health facility registry	DepEd clinic		Public	Level 1
	Provincial health office		Public	Level 1
	Rural health unit		Public	Level 1
	Barangay health station		Public	Level 0
	Psychiatric care facility		Private	Level 1
	Hospital		Both	Level 2
	Infirmary		Both	Level 2
	Birthing home		Both	Other

Abbreviations: DHS, Demographic and Health Survey; DOTS, directly observed treatment, short-course; ITIS, integrated TB information system; TB, tuberculosis.

**Figure 1.**
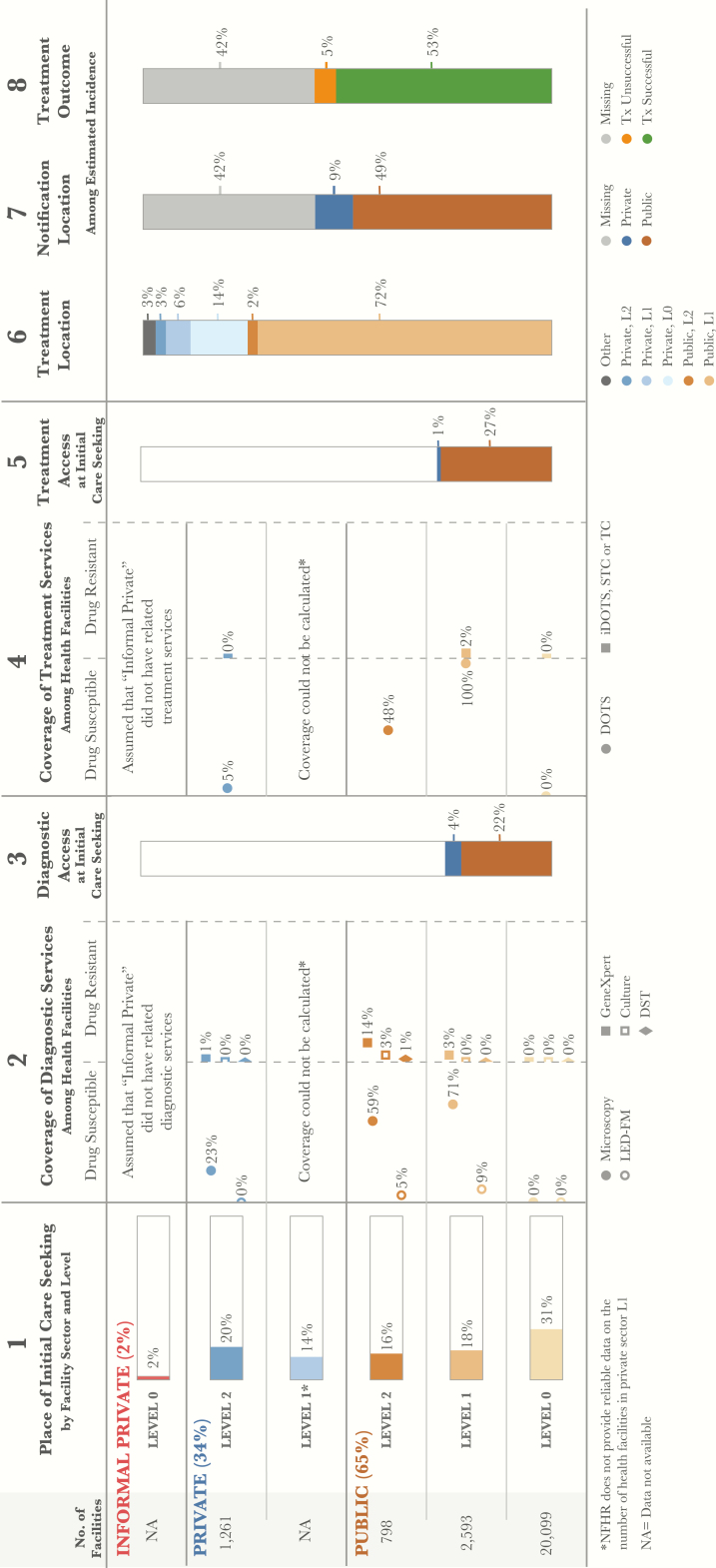
Patient pathway visual—national level. The patient pathway describes the care seeking patterns of patients and how those patients may intersect with TB services. Column 1 starts by showing the sectors and levels of the health system (note: sectors and levels where no data was available are excluded from the pathway). The percent next to each sector title is the share of patients who initiate care seeking in this sector [6]. The next part of column 1 shows the reported number of health facilities at each sector and level. These were used as the denominator for calculations in columns 3 and 5 [18]. Outside of birthing facilities, there was no data on the number of health facilities in the private sector level 1; this level was excluded from the analysis. Finally, column 1 shows the percentage of patients who sought initial care seeking advice or treatment in a health facility [6]. Column 2 shows the percentage of health facilities that have diagnostic tests across each sector and level of the health system (i.e., coverage). Diagnostic tools are separated by tools for diagnosing drug-sensitive TB (DS-TB) and those diagnosing drug-resistant TB (DR-TB). Coverage in column 2 was calculated using the health facilities in column 1 as the denominator and a list of health facilities with TB services provided by the national TB program as the numerator [17, 18]. This list included both public and private facilities. Column 3 shows the estimated percentage of patients likely to access a facility with TB diagnosis available on their initial visit to a health care facility. This column was calculated by multiplying the share of care seeking at each sector/level of the health system by the coverage of microscopy at each respective sector/level and summing the total. Columns 3 and 5 separate public and private sectors based on each sector’s contribution to TB services access at initial care seeking. Column 4 shows the percentage of health facilities that have TB treatment available at each sector and level of the health system (i.e., coverage). Coverage of treatment services was calculated using data provided by the NTP about facilities that have TB treatment services available [17, 18]. Similar to the diagnosis coverage in column 2, treatment coverage separates those facilities that provide treatment for drug-sensitive TB (DS-TB) and those that provide treatment for drug-resistant TB (DR-TB). Column 5 shows the estimated percentage of patients likely to access a facility with TB treatment available on their initial visit to a health care facility. This column was calculated by multiplying the share of care seeking at each sector/level of the health system by the coverage of first-line treatment (DOTS) at each respective sector/level and summing the total. Column 6 shows the location of treatment among participants in the 2016 prevalence survey who had previously received TB treatment, the assumption being that this metric provides the most representative picture of where patients are treated nationwide [16]. Column 7 shows which sector provided case notification and is calculated as a share of the overall estimated incidence in 2015 [13]. Column 8 shows the treatment outcome of notified cases among the overall estimated incidence for 2015 [13]. Columns may not add to 100%, due to rounding. For more details on the data sources used in the pathway, see supplementary materials. Abbreviations: DOTS, directly observed treatment, short-course; NTP, National TB Program; TB, tuberculosis.

At the national level, 2 separate analyses of care initiation were performed using different data sources; the first analysis was conducted with regard to general care seeking patterns and second analysis was conducted specifically for presumptive TB cases. The parallel analyses were completed to validate the use of general care seeking data as a proxy for TB-specific care seeking, given that the DHS sample—which reported data only on general care-seeking patterns—enabled subnational analysis, whereas the prevalence survey—which included specific care-seeking data for presumptive TB cases—did not. The 2013 DHS provided data about facilities where respondents sought general care in the 30 days prior to the survey. These estimates are shown in column 12 of the DHS patient pathway visual (Figure 1) [6]. The second national PPA was completed using the NTPS data. The NTPS asked participants where they initiated care when they first developed TB symptoms [16]..

The PPA using NTPS data did not include care seeking at the community (Barangay) level. However, the DHS data found that 31% of patients initiated care at public Barangay Health Stations (BHSs). This discrepancy is due to different classifications of health facilities in the two surveys. Because the NTPS did not include BHSs in its facility list, patients who initiated care at BHSs were identified as having initiated care at L1 facilities. In addition, TB DOTS centers were designated as public L1 facilities in the NTPS, whereas a portion of these clinics may have been classified as public TB DOTS hospitals in the DHS. Taking these differences into account, the care seeking approach of patients with TB symptoms in the NTPS resembled the approach taken by those with general symptoms in the DHS. Thus, data from DHS could be utilized to understand a TB patient’s journey within the Filipino health system. Given the larger sample size, general care seeking data from the DHS were used for all subnational analyses.

To estimate the availability of TB services in each health facility, a national electronic TB register (*integrated TB information system* [ITIS]) was combined with the national health facility registry (NHFR) [17, 18]. The ITIS provided a list of all facilities in the country that had TB diagnostic and treatment services. The NHFR provided a list of all health facilities that were registered within the country. It was the most comprehensive list available of all health facilities in the country and included detailed information across both the public and the private sector. Similar to the care seeking data, each of these data sources used different naming conventions for each type of health facility, so again each health facility was designated to standardized categories outlined in Table 2.

After categorizing each health facility and eliminating those facilities where respondents could not have sought TB care (e.g., birthing homes), there were no facilities listed that could be categorized as L1 private facilities, so TB service alignment for this category of facility could not be calculated. This is an important limitation, given that the recent prevalence survey suggests that nearly 20% of TB patients received treatment in private sector nonhospital facilities (i.e., L0 and L1). Thus, the PPA likely underestimates the percent of patients who can receive TB services (diagnosis and treatment) at their first visit to the health system, given that many patients can and do eventually receive care in the private sector [16].

To determine diagnostic coverage, access to the following types of diagnostic tools was measured: Microscopy (DSSM and LED-FM), GeneXpert, Culture (TBC), and Drug Sensitivity Testing (DST) [17]. To calculate diagnostic coverage for each sector and level, data on the number of health facilities with a given TB diagnostic service was divided by the total number of health facilities within that category. These estimates are shown in column 2 of the patient pathway visual (Figure 1).

To calculate the percentage of TB patients accessing diagnostic services at the point of care initiation, the proportion of patients who sought care at each health sector and level was multiplied by the coverage of TB diagnostic services in that category. This calculation was made for each health facility sector and level. For reasons discussed above, private sector level 1 was excluded from this calculation. The results were summed to provide an estimate of the accessibility of TB diagnostics upon care initiation. Because there were several types of diagnostic services available, facilities with either microscopy or Xpert were included. These estimates are shown in column 3 of the patient pathway visual (Figure 1).

Column 4 (Figure 1) shows the coverage of TB treatment services at each facility level and sector. Two types of treatment sites were included in the calculation: facilities with capacity to manage drug-sensitive TB treatment and MDR-TB treatment sites [17]. The same method used to determine diagnostic availability was used to determine the coverage of treatment by level and sector. In some regions, there were a greater number of health facilities listed that had TB services than there were health facilities in the NHFR. This was due to the inclusion of various categories of service providers, such as DOTS, iDOTS, satellite treatment centers, and treatment centers. In regions where all facilities had at least one category of TB treatment service, coverage was listed as 100%.

Column 5 represents the likelihood of a patient initiating care in a facility with any TB treatment services available. The calculations were performed in the same way as those for column 3.

Column 6 shows data from the most recent TB prevalence survey on the location of treatment for those survey participants who were currently on treatment or had previous treatment for tuberculosis since 2011 [16].

Column 7 shows which sector was the source of those cases notified to the NTP in 2015 [13]. Notifications were calculated as a share of the estimated burden in 2015 [13]. Finally, column 8 shows the treatment outcomes for notified cases as a percentage of the overall estimated burden. Both columns 7 and 8 include the share of cases that are missing or not notified and thus the source of treatment and outcome of treatment are unknown.

### Limitations

There are several important limitations to the PPA as described above. Most importantly, the lack of data on level 1 private sector facilities makes a full interpretation of private sector alignment challenging. Given that the recent prevalence survey suggests that more than a quarter of patients are treated outside of the public sector, gaining additional insight and data on private sector care, including the number of facilities, is crucial. A second limitation is the use of the national facility health registry (NFHR) as the denominator for coverage estimates. Although this is the best available data on the number of facilities in the country, the list may underreport facilities across the country, which could lead to overestimation of coverage at each level. Finally, the care seeking data sources did not provide information about care seeing in private pharmacies. The prevalence survey results reported that 14% of patients received treatment from L0 private facilities (pharmacies) (Figure 1, column 6), suggesting that at some point in their journey, patients are seeking care in pharmacies [16]. It would be instructive to explore the extent to which pharmacies are used as a location for initial care seeking or are limited only to treatment of patients once they have sought care elsewhere. Further limitations of the PPA methodology are described in more detail elsewhere [15].

## RESULTS

### Differences in Diagnostic and Treatment Access at National and Subnational Levels

At the national level, DHS data suggest that 65% of patients initiated care in the public sector, with the remainder initiating care in the private sector (Figure 1) [6]. Despite the focus on expanding access to TB diagnostics, sputum microscopy was available in only 59% of provincial health offices, their adjoined health facilities, and regional hospitals (at L2), and 71% of rural health units (RHUs) or health centers (at L1) (Figure 1, column 2). Furthermore, the DHS data estimated that nearly a third of patients initiated care at BHSs, which provide primary care services at community level [6] (Figure 1, column 1). None of the BHSs had microscopy or other diagnostic technologies, although some did have sputum collection or smear and transport capacity. However, coverage data for these services were not available. By comparison, 23% of private hospitals (at L2) had smear microscopy. Overall, the national PPA suggests that only 26% of patients had access to sputum microscopy at the location of care initiation (Figure 1, column 3).

The subnational PPAs identified important regional differences in care seeking and diagnostic accessibility. Given the much higher rates of microscopy coverage in the public sector compared with the private sector, regions with higher rates of care initiation in the public sector—at RHUs and public hospitals—had a higher level of diagnostic coverage than regions with higher rates of care initiation in the private sector. Nationally, the share of presumptive TB patients who accessed diagnostic services at the location of care initiation was also influenced by regional variations in the percentage of patients initiating care at BHSs (L0), as well as by the availability of microscopy at the RHU level (L1). In 9 regions, more than one-third of patients initiated care at BHSs. In 6 regions, microscopy coverage at the RHU level exceeded 95%.

**Figure 2.**
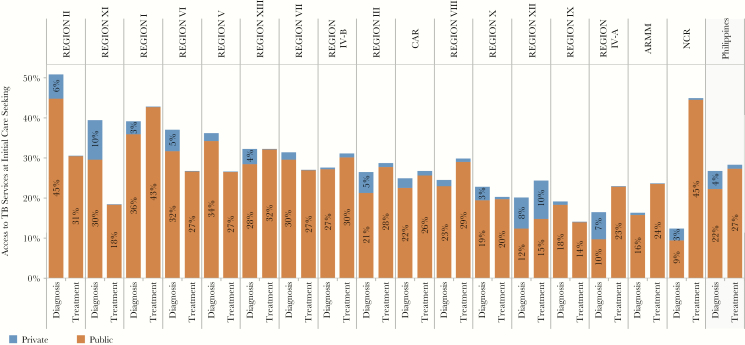
Access to TB services at initial care seeking—by region. The patient pathway analysis was completed at the national level as well as subnationally for 17 regions. The figure shows the Access to Diagnosis and Access to Treatment at Initial Care Seeking metrics across each of these regions (columns 3 and 5 of the patient pathway visual; see Figure 1). Abbreviation: TB, tuberculosis.

At the national level, only 28% of patients initiated care in a facility that had TB treatment available (Figure 1, column 5). Because 100% of RHUs were able to provide TB treatment for DS-TB, regions in which more patients sought care at RHUs were more likely to provide TB treatment services at the point care initiation, for example, 45% in NCR and 42% in Region I. By contrast, in regions where few patients initiated care at RHUs, patients were less likely to access treatment where they initiated care, for example, 18% in Region XI and 23% in Region IV-A (Figure 2).

At the subnational level, diagnostic and treatment access varied widely between regions (Figure 2). Figure 2 shows the availability of TB services in both the public and private sectors combined. Only Regions I, II, and XIII had relatively high coverage of both diagnostic and treatment services at health facilities where patients initiated care. For the majority of regions, the alignment between the location of care initiation and the availability of TB diagnostic and treatment services was not as strong. Some regions had high diagnostic availability and low treatment availability at the location of care initiation, whereas others had low treatment availability with high access to diagnostic services, for example, Regions VII, VI, XI.

### Patients Accessing Private Sector for Care had Lower Access to TB Diagnostics and Treatment

Although 36% of patients initiated care in the private sector, the NTP reported that only 7% of all notified cases came from the private sector. Although the NTP indicated that more than 72% of private hospitals reported some TB patients in 2016, programmatic supervision and qualitative reports suggest that not all TB patients cared for in private hospitals are reported. In 12 of the 17 regions, between 25% and 40% of care was initiated in the private sector. The NTP accredits private facilities that have appropriate diagnostic and treatment practices, as part of engaging these providers in the national health insurance network. The numerous stand-alone private clinics were unlikely to have been captured in the NHFS data, and because it was unknown if these independent private clinics had TB services, the accessibility of TB services in the private sector may have been higher than estimated by the PPA.

There were major differences in diagnosis and treatment at the subnational level. In Regions II and XII, NTP-accredited microscopy services were available in 45% and 49% of private hospitals, respectively, but in 11 of 17 regions, less than 25% of these hospitals had been accredited for TB diagnosis. The private sector was also lacking in documented capacity for TB treatment. All regions except one had poor availability of NTP-accredited TB treatment services, with accredited TB treatment available in <7% of private hospitals. Regions with high rates of care initiation in the private sector (e.g., 44% in Region III, 40% in Region IV-A and CAR) had lower overall access to NTP-accredited TB diagnostic and treatment services for all patients (Figure 2).

### Limited Access to MDR-TB Diagnosis and Care

Based on the 2013 DHS data, Xpert was available nationally in 14% of public hospitals and 3% of RHUs. At the subnational level, Xpert coverage in public hospitals ranged from 37% in NCR region to 7% in Region V. Xpert was available in <9% of RHUs and was available in only a small number of private facilities. The NTP recognized the important role of Xpert in screening for drug resistance, and the number of Xpert sites has been scaled up from 84 in 2014 to 180 in 2016. Until recently, the country utilized a centralized model of MDR-TB care. This helps to explain the fact that 10% of public hospitals could provide care for MDR-TB patients, but only 2% of RHUs had that same capability (Figure 1, column 2). The availability of MDR-TB treatment varied modestly between regions, with none exceeding availability in 20% of public hospitals.

## DISCUSSION

Results of the recently completed national TB prevalence survey bring a sense of urgency to the interpretation and use of the results PPA to strengthen access to TB care. With a burden of tuberculosis that is nearly 70% higher than previously estimated, the Philippines must do more to reach the estimated 42% of DS-TB patients who are currently not notified, as well as the 83% of MDR-TB patients for whom there is no diagnosis and treatment information.

### Need to Better Understand the Size and Practices of the Private Sector to Plan for Private-Public Cooperation

This PPA revealed that although just over one-third of TB patients initiate care in the private sector, only 16% of all TB cases notified to the NTP came from the private sector. This represents a major drop-off between care initiation and diagnosis, notification, and treatment. In fact, this finding alone could explain what happens to most of the missing cases in the Philippines. If most of the missing TB patients initiated care in the private sector but were never notified, it is possible that they were not successfully treated. Based on previous studies on the sale of anti-TB medicines in the private sector, it appears plausible that many of the missing TB patients are indeed receiving treatment in the private sector. A study by Garfin and Islam found that the quantity of anti-TB medicine sold in the private sector each year was sufficient to treat 80% of the estimated number of TB patients in the Philippines [19]. However, there are 2 factors that make this conclusion unsatisfactory. First, the PPA points to the limited availability of diagnostic and treatment services in private facilities. Given this limited capacity as well as the recent finding that the prevalence rate is not declining, the quality of care in many private facilities is likely poor [16]. The second consideration relates to the likely overdiagnosis of tuberculosis in both the public and private sectors, given an overreliance on x-ray [4, 13]. It has been estimated that as many as a quarter of the notified cases are false-positives [4]. Taking this into account, the number of missing cases of tuberculosis is considerably higher and not all can be accounted for in the private sector.

Regardless of what currently happens to patients who initiate care in the private sector, it is clear that TB patients would benefit from an expansion of quality-assured TB services in private sector facilities. A major limitation to developing a private sector engagement program is the paucity of data regarding TB service availability practices in the private sector. Private clinics, in particular, are largely unregulated. It is likely that many more private providers than are captured within the National Health Facility Registry operate within the Philippines. Similarly, private providers are known to deliver TB services that are not included in the government’s national recording and reporting system for tuberculosis, ITIS. The NTP plans to undertake an inventory study to determine how many private clinicians are engaged in TB care and what services they offer. In addition, a national law passed in 2016, known as “the TB Law,” requires the notification of all new TB cases—in any sector—to the NTP. Although enforcement mechanisms will need to be instituted, the law is a step toward unifying the information flow between the disparate public and private health systems.

### Misalignment in the Public Sector Can be Addressed Strategically Through the New National Plan

The PPA has identified gaps in the public sector TB diagnostic and treatment network that could also account for some of the missing cases. Although smear microscopy is available in most RHUs and public hospitals, the availability of microscopy in these facilities is far from complete; 30–40% of RHUs and public hospitals still do not have access to smear microscopy. Furthermore, a third of all patients initiate care at BHSs where diagnostic services for TB are unavailable. Finally, at the subnational level, there is still significant misalignment between the availability of diagnostic technologies and the availability of treatment. Given all of these gaps, even some patients who initiate care in the public sector may not immediately receive an appropriate diagnosis and treatment.

Although the PPA is primarily focused on the availability of microscopy, the future of TB diagnosis in the Philippines will be based on molecular testing. The new national strategic plan for tuberculosis envisages the use of Xpert for 100% of presumptive TB patients by 2020, gradually replacing sputum microscopy. However, simply replacing microscopy with Xpert will not guarantee equitable and timely access to diagnosis in the public sector. Recognizing this, the country plans to introduce a robust sputum transport network by 2018. The sputum transport network will be strategically designed to optimize, but not overload, the expanded number of Xpert machines. All public facilities will be networked to specific Xpert machines so as to minimize delays and cost for patients, while maximizing the efficiency of all equipment.

Given the findings of the PPA, a logical extension of the planned referral network would involve the inclusion of private providers into the Xpert referral network. In the new national strategic plan, the NTP aims to engage 80% of all private care providers. The widespread use of Xpert could help address the overreliance on x-ray in the private sector and treatment based on clinical diagnosis alone. Some private providers have already purchased Xpert equipment, although the cost of Xpert testing to patients is currently approximately 160 USD per test, a prohibitively high cost for many Filipino patients.

The existing spoke and hub model that links BHSs in the communities to RHUs can be used as a model for the sample referral network, ensuring that sample referral extends directly to patients where seek care. With nearly a third of presumptive TB patients initiating care at the community level, this linkage must be systematized. Currently, only a few BHSs have remote sputum smearing stations or sputum collection and transport stations. Lessons learned from successful models can be rapidly expanded and incorporated into the Xpert referral network. Furthermore, L0 is a critical level for expanding treatment availability for both DS- and DR-TB. The World Health Organization recently issued revised guidelines for the programmatic management of DS-TB, which advocates strongly for community-based care. Community care makes sense in the Philippines, and based on the findings of the PPA, may be an important step toward finding the missing TB cases and providing patient centered care.

### The Approach to MDR-TB Can Follow the Lead of Patient-centered DS-TB Care

MDR-TB care has scaled up slowly in the Philippines. The efforts, described above, to introduce Xpert as the initial test for all presumptive TB patients will certainly identify more TB patients requiring MDR-TB treatment. The low treatment success rates (50%) for MDR-TB patients are often blamed on the centralized care model that requires patients to be treated far away from their families and communities for extended periods of time. Based on the results of the PPA, it is clear that to provide patient-centered care, support for daily treatment needs must be available at L0 and L1 facilities. Enablers or other innovations may be needed to ensure that patients receive their monthly clinical and laboratory monitoring in a patient-centered manner as well.

The new strategic plan explores community-based DOTS for MDR-TB patients. This program will be designed using a hub and spoke model to support lower level health staff with the provision of MDR-TB care. A national Nurse Deployment Program (NDP) is bringing more clinical care capacity to local levels, enabling treatment with even the more complex second-line regimens, including injectables. In BHSs without clinically qualified staff to administer injections, linkages with local birthing homes may offer access to health staff that can provide injections to MDR-TB patients on weekends—and possibly even on weekdays—so that they don’t have to travel to distant RHUs and city hospitals.

## Supplementary Data

Supplementary materials are available at *The Journal of Infectious Diseases* online. Consisting of data provided by the authors to benefit the reader, the posted materials are not copyedited and are the sole responsibility of the authors, so questions or comments should be addressed to the corresponding author.

## Supplementary Material

Supplementary Material

## Notes

***Supplement sponsorship.*** This work is part of a supplement sponsored by the Bill & Melinda Gates Foundation.

***Potential conflicts of interest.*** All authors: No reported conflicts of interest. All authors have submitted the ICJME Form for Disclosure of Potential Conflicts of Interest. Conflicts that the editors consider relevant to the content of the manuscript have been disclosed.
