# Supplementary material for: Using Patient Pathway Analysis to Design Patient-centered Referral Networks for Diagnosis and Treatment of Tuberculosis: The Case of the Philippines
Source: J Infect Dis. 2017 Nov 6;216(Suppl 7):S740–7. doi: 10.1093/infdis/jix391 (PMC5853338; doi:10.1093/infdis/jix391)
Supplement: Supplementary Material [file jix391_suppl_supplementary-material.docx]

| **Country: Philippines** | | | | | |
| --- | --- | --- | --- | --- | --- |
| **Data Source** | **Survey Type** | **Survey Question** | **Reported Metric** | **Sample Size** | **PPA Step** |
| National Health Facility Registry (accessed 04/01/17) | NHFR is most comprehensive list of health facilities in the Philippines  <http://nhfr.doh.gov.ph/rfacilities2list.php> | For each facility, the NFHR provides:   - Location - Facility Type - Facility Staff Head - Ownership (e.g. public, private) | N/A | Government (public sector) facilities in database: 24,118  Private sector facilities in database: 2,061 | Number of Health Facilities |
| 2013 Demographic and Health Survey | Stratified two-stage sample designed to provide data representative of  the country and its 17 administrative regions. For the 2013 NDHS sample, 16,732 households were selected, of which 14,893 were occupied. Of these households, 14,804 were successfully interviewed, yielding a household response rate of 99.4%. | Question 206. In the last 30 days, has any member of your household visited a health facility for consultation/advice or treatment anywhere?  Question 210. Where was consultation/advice or treatment first sought for (NAME IN 208)’s illness/injury/check-up/laboratory?   - Public sector: Regional hosp./public med ctr; Provincial hosp; District hospital; Municipal Hosp; RHU/Urban Hlth ctr./Lying-in; Barangay Hlth St; Mobile clinic; Other public - Private sector: Private hosp./clinic; Lying-in clinic/birthing home; Private clinic; Private pharmacy; Mobile clinic; Other private - Alternative medical: Hilot/herbalists; Therapeautic massage center; Other alternative healing - Not medical sector: shop selling drugs/market; Faith healer; Other | Initial care seeking location first  visited by persons who sought advice or treatment in the 30 days preceding the survey.  (Table 13.4, p.164) | n=7482  Number of households that included a member who visited a health facility for consultation/advice or treatment within the last 30 days | Step 1 –  Initial care seeking patterns |
| Philippines ITIS Database (accessed 04/01/17) | Database spreadsheet provided by NTP | The database from the NTP provided a list of diagnosis and treatment facilities across the country in both the public and private sectors. For each facility, the database provides:   - Location - Facility Name   Facility Type (e.g. hospital, clinic, etc). | N/A | Diagnostic Facilities: 2796  Treatment Facilities: 3662 | Steps 2 and 4 – TB services coverage |
| 2017 National TB Prevalence Survey | Preliminary data shared from NTP for use in PPA case study. Full prevalence study report not yet available at time of publication. | N/A | Actions related to survey participants who are currently being treated for TB or treated in 2011 or beyond: Source of medicines | Survey participants with response to question about TB history:   - No treatment history – 43,956 - Currently on treatment – 170 - With treatment after 2011 – 2,563   Participants with treatment after 2011, number who provided source of medicines – 1,211 | Step 6 – Treatment location |
| 2016 WHO Global TB Report | Annual report providing data on TB epidemiology, health systems and financing for 194 member state.  Notification location data available in table 4.2 of annual report.  Raw data accessed via Global TB Database available here:  <http://who.int/tb/data/en/> | N/A | Estimated burden – 324,000 cases  TB treatment coverage (case detection rate) – 85%  New and relapse notified cases –  276,672  Share of notified cases from private sector – 6.4%  Treatment success rate – 92% | | Step 7 –  Among estimated burden-notification source  +  Step 8 –  Among estimated burden-successfully treated cases |
